# On the Kinetic and Allosteric Regulatory Properties of the ADP-Glucose Pyrophosphorylase from *Rhodococcus jostii*: An Approach to Evaluate Glycogen Metabolism in Oleaginous Bacteria

**DOI:** 10.3389/fmicb.2016.00830

**Published:** 2016-06-02

**Authors:** Antonela E. Cereijo, Matías D. Asencion Diez, José S. Dávila Costa, Héctor M. Alvarez, Alberto A. Iglesias

**Affiliations:** ^1^Laboratorio de Enzimología Molecular, Instituto de Agrobiotecnología del Litoral, CONICET, Centro Científico Tecnológico, Facultad de Bioquímica y Ciencias Biológicas, Universidad Nacional del LitoralSanta Fe, Argentina; ^2^Centro Regional de Investigación y Desarrollo Científico Tecnológico, Facultad de Ciencias Naturales Universidad Nacional de la Patagonia San Juan BoscoComodoro Rivadavia, Argentina

**Keywords:** actinobacteria, carbohydrate–lipids metabolisms, allosteric regulation, glucosamine-1P, ADP-glucose pyrophosphorylase

## Abstract

*Rhodococcus* spp. are oleaginous bacteria that accumulate glycogen during exponential growth. Despite the importance of these microorganisms in biotechnology, little is known about the regulation of carbon and energy storage, mainly the relationship between glycogen and triacylglycerols metabolisms. Herein, we report the molecular cloning and heterologous expression of the gene coding for ADP-glucose pyrophosphorylase (EC 2.7.7.27) of *Rhodococcus jostii*, strain RHA1. The recombinant enzyme was purified to electrophoretic homogeneity to accurately characterize its oligomeric, kinetic, and regulatory properties. The *R. jostii* ADP-glucose pyrophosphorylase is a homotetramer of 190 kDa exhibiting low basal activity to catalyze synthesis of ADP-glucose, which is markedly influenced by different allosteric effectors. Glucose-6P, mannose-6P, fructose-6P, ribose-5P, and phospho*enol*pyruvate were major activators; whereas, NADPH and 6P-gluconate behaved as main inhibitors of the enzyme. The combination of glucose-6P and other effectors (activators or inhibitors) showed a cross-talk effect suggesting that the different metabolites could orchestrate a fine regulation of ADP-glucose pyrophosphorylase in *R. jostii*. The enzyme exhibited some degree of affinity toward ATP, GTP, CTP, and other sugar-1P substrates. Remarkably, the use of glucosamine-1P was sensitive to allosteric activation. The relevance of the fine regulation of *R. jostii* ADP-glucose pyrophosphorylase is further analyzed in the framework of proteomic studies already determined for the bacterium. Results support a critical role for glycogen as a temporal reserve that provides a pool of carbon able of be re-routed to produce long-term storage of lipids under certain conditions.

## Introduction

*Rhodococcus jostii* RHA1 is a soil bacterium with the ability to synthesize and accumulate significant amounts of triacylglycerol (TAG) during cultivation of cells under nitrogen-limiting conditions (Hernandez et al., 2008). In addition, this strain produces glycogen in low amounts (equivalent to 2–3% of cellular dry weight), mainly during exponential growth phase, which seems to be a common feature among rhodococci (Hernandez and Alvarez, 2010). The industrial relevance of bacterial TAG as a source of biofuels, biolubricants, or oleochemicals promoted basic and applied research on oleaginous actinobacteria. Knowledge about the biochemistry of *Rhodococcus* spp. still fragmentary although some advances have been made in the last years, principally in model oleaginous rhodococci such as *R. jostii* RHA1 and *Rhodococcus opacus* PD630. Advances were achieved using high-throughput -omics and from functional characterization of assorted genes/proteins of the cellular metabolism. To improve our understanding of metabolism in *R. jostii* RHA1, we recently performed an extensive proteomic study of this oleaginous bacterium under conditions of TAG accumulation (Davila Costa et al., 2015). We observed extensive metabolic remodeling, involving carbon flux redirection toward TAG synthesis, with glycolysis mainly providing precursors for lipogenesis. In this context, a deeper knowledge on the relationship between pathways such as glycolysis, glycogen metabolism, and lipogenesis in this bacterium, as well as on the processes to ensure sufficient carbon supply for lipid anabolism, may contribute to delineate the metabolic map for rhodococcal cells.

Glycogen is a polysaccharide composed of glucose (Glc) units in an α-1,4-linked linear arrangement with α-1,6-branches (Ballicora et al., 2003, 2004; Preiss, 2009). Although, the particular physiological role of this biopolymer in bacteria has not been clearly established, it was suggested that its accumulation could be advantageous during starvation periods, providing a stored source of energy and carbon surplus (Ballicora et al., 2003). However, in certain microorganisms glycogen may have a role as metabolic intermediate, since it is accumulated mainly during exponential growth and degraded during the stationary phase. Consequently, the polysaccharide has been proposed as a “carbon capacitor” for glycolysis during exponential growth (Seibold and Eikmanns, 2007; Seibold et al., 2007, 2011). Glycogen synthesis involves elongation of an α-1,4-glycosidic chain by glycogen synthase (EC 2.4.1.21; GSase), using ADP-glucose (ADP-Glc) as the glucosyl donor (Ballicora et al., 2003; Preiss, 2009). The key regulatory step in bacterial glycogen metabolism occurs at the level of ADP-Glc synthesis, in the reaction catalyzed by allosteric ADP-Glc pyrophosphorylase (EC 2.7.7.27; ADP-Glc PPase; Ballicora et al., 2003, 2004). ADP-Glc PPase and GSase are respectively coded by *glgC* and *glgA* which, together with *glgB* (the gene coding for branching enzyme), constitute the classical GlgCA pathway for glycogen synthesis in prokaryotes (Chandra et al., 2011). This pathway is finely regulated at the level of ADP-Glc PPase by key metabolites of the main carbon route of the respective organism (Ballicora et al., 2003, 2004). Recently, it has been shown the existence of an alternative pathway for glycogen synthesis in actinobacteria, which involves the enzyme known as GlgE (Chandra et al., 2011). *R. jostii* RHA1 possess the complete set of genes for glycogen metabolism (Hernandez et al., 2008), and a current challenge is to determine how the synthesis of the polysaccharide is regulated in an oleaginous prokaryote. The available genomic and proteomic information prompted us to characterize the kinetic and regulatory properties of recombinant ADP-Glc PPase from *R. jostii* as a way to gain information about its physiological role in bacteria.

## Materials and Methods

### Chemicals

Protein standards, antibiotics, IPTG, Glc-1P, Glc-6P, glucosamine-1P (GlcN-1P), galactose-1P (Gal-1P), *N*-acetylglucosamine-1P (GlcNAc-1P), 6-phosphogluconate (6-PGlcA), ATP, UTP, GTP, CTP, fructose-6P (Fru-6P), ribose-5P (Rib-5P), phospho*enol*pyruvate (PEP), pyruvate (Pyr), NADPH, and oligonucleotides were obtained from Sigma-Aldrich (St. Louis, MO, USA). All other reagents were of the highest quality available.

### Bacteria and Plasmids

*Escherichia coli* Top 10 F′ cells (Invitrogen) and pGEM^®^T Easy vector (Promega) were used for cloning procedures. The *glgC* gene from *R. jostii* (*RjoglgC*) was expressed in *E. coli* BL21 (DE3; Invitrogen) using pET28c vector (Novagen). Alternatively, *RjoglgC* was expressed in *E. coli* AC70RI-504 using pMAB5 vector as stated before (Iglesias et al., 1993; Asencion Diez et al., 2013a). DNA manipulations, *E. coli* cultures as well as transformations were performed according to standard protocols (Sambrook and Russell, 2001).

### Gene Amplification

The *glgC* gene (ID 4223526) coding for ADP-Glc PPase from *R. jostii* RHA1 was amplified by PCR using genomic DNA as template. Primers were designed according to available genomic information (McLeod et al., 2006) in the GenBank database^1^. The forward primer (5′-CATATGAGGAGCCAGCCACATGTG-3′) introduced an *Nde*I site (underlined) overlapping the translational initiation codon, which was changed from GTG to ATG. The reverse primer introduced a *Sac*I site (underlined) downstream the stop codon: 5′-GAGCTCTAGATCCAGACGCCCTTGC-3′. PCR reaction mixtures (50 μl) contained 100 ng of genomic DNA, 50 pmol of each primer; 0.2 mM of each dNTP; 2.5 mM Mg^2+^, 8% (v/v) DMSO and 1 U *Pfu* DNA polymerase (Fermentas). Standard conditions of PCR were used for 30 cycles: denaturation at 94°C for 1 min; annealing at 60°C for 42 s, and extension at 72°C for 3 min, with a final extension of 10 min at 72°C. PCR reaction mixtures were defined in 1% (w/v) agarose gel and purified by means of Wizard SV gel and PCR Clean Up kits (Promega). The amplified gene [previously treated with *Taq* polymerase (Fermentas) and dATP] was cloned into the T-tailed plasmid pGEM-TEasy. The identity of the cloned gene was determined by full sequencing.

### Cloning Procedures

Gene identity was confirmed by DNA sequencing (Macrogen, South Korea). Afterward, the pGEMTEasy plasmid harboring *glgC* coding sequence was digested with *Nde*I and *Sac*I and the released gene was cloned into pET28c and pMAB5, to obtain the expression vectors [pET28c/*RjoglgC*] and [pMAB5/*RjoglgC*], respectively.

### *R. jostii* ADP-Glc PPase Expression in *E. coli*

*Escherichia coli* AC70RI-504 cells lacking endogenous ADP-Glc PPase activity were transformed with [pMAB5/*RjoglgC*]. Transformed cells were grown in 1 l of LB medium (10 g/l tryptone; 5 g/l yeast extract; 10 g/l NaCl) at 37°C, 200 rpm, until OD_600_ of ∼1.1. Recombinant protein expression was induced with 0.4 mM IPTG for 20 h. As well, competent *E. coli* BL21 (DE3) cells harboring pGro7 plasmid (Takara) were transformed with the [pET28c/*RjoglgC*] construction. Protein expression was carried out using YT2X medium (5 g/l tryptone; 16 g/l yeast extract; 5 g/l NaCl) supplemented with 100 g/ml each kanamycin and chloramphenicol. Cells were grown at 37°C and 250 rpm until OD_600_ ∼0.6. Chaperones and recombinant gene expression were induced for 16 h at 20°C by the addition of 0.5 mg/ml L-arabinose and 0.5 mM IPTG, respectively. After induction, cells were harvested by centrifugation at 5000 × *g* for 10 min and stored at -20°C until use.

### Purification of the Recombinant Protein

Purification procedures were done at 4°C. Cells were harvested by centrifugation at 5000 × *g* for 10 min, resuspended in *Buffer H* [50 mM Tris-HCl pH 8.0, 300 mM NaCl, 5% (v/v) glycerol] and disrupted by sonication on ice (5 s pulse on with intervals of 3 s pulse off for a total time of 15 min). The suspension was centrifuged twice at 30000 × *g* for 10 min and the supernatant (crude extract) was loaded in a 1 ml HisTrap column (GE Healthcare) previously equilibrated with *Buffer H*. The recombinant protein was eluted with a linear gradient from 10 to 240 mM imidazole in *Buffer H* (120 volumes), and fractions containing the highest activity were pooled and concentrated to 2 ml. Active ADP-Glc PPase fractions were dialyzed against *Buffer X* [50 mM HEPES pH 8.0, 0.1 mM EDTA, 0.5 mM DTT, 20% (w/v) sucrose]. Under these conditions the enzyme was stored at -80°C until use, being fully actives for at least 3 months.

### Protein Methods

Protein concentration was determined by the modified Bradford assay (Bradford, 1976) using BSA as a standard. Recombinant proteins and purification fractions were defined by sodium dodecyl sulfate polyacrylamide gel electrophoresis (SDS-PAGE) according to (Laemmli, 1970). Gels were loaded with 5–50 μg of protein per well and stained with Coomassie Brilliant Blue. Western blotting was performed after standard techniques (Sambrook and Russell, 2001). Proteins in the gel were blotted onto PVDF membranes using a Mini-PROTEAN II (Bio-Rad) apparatus. The membrane was blocked 2 h at room temperature and subsequently incubated overnight with primary antibody at 4°C. Then, membranes were incubated with rabbit anti-IgG conjugated to peroxidase (Sigma-Aldrich) during 1 h at 25°C. Detection was carried out with 3,3-diaminobenzidine and hydrogen peroxide in 50 mM Tris-HCl, pH 8.0, 150 mM NaCl. Antibody against *Mycobacterium tuberculosis* ADP-Glc PPase was produced in our lab according to established methods (Vaitukaitis, 1981) and used as primary antibody. It was purified from rabbit sera by consecutive precipitation steps with ammonium sulfate 50 and 33% (twice) saturation solutions. After that, antibody was resuspended in TBS buffer (Tris-HCl pH 8.0, NaCl 150 mM) and desalted using an ultrafiltration device with a 30 kDa cut-off (Amicom).

### Iodine Staining Assay

Experiments were carried out according to previous reports (Asencion Diez et al., 2013a; Demonte et al., 2014). Briefly, transformed *E. coli* AC70R1-504 cells with [pMAB5/*RjoglgC*] plasmid were inoculated onto 3 ml of LB medium and growth at 37°C until they reached an OD_600_ ∼0.8. Then, protein expression was induced with 0.5 mM IPTG for 3 h at 20°C. Afterward, Glc was added to a final concentration of 2% (w/v) and the incubation was extended for 1 h. An aliquot of 0.1 ml was withdrawn and centrifuged in a 1.5 ml microcentrifuge tube at 10000 × *g* for 5 min. Supernatant was removed, leaving the compact pellet at the bottom of the tube. Then, the tube was turned upside down and an iodine crystal positioned in the cap to stain the cell pellet with iodine vapor (Demonte et al., 2014).

### Enzyme Activity Assays

ADP-Glc PPase activity was determined at 37°C in ADP-Glc synthesis direction by following the formation of P_i_ (after hydrolysis of PP_i_ by inorganic pyrophosphatase) by the highly sensitive colorimetric method previously described (Fusari et al., 2006). The reaction mixture contained 50 mM MOPS pH 8.0, 5 mM MgCl_2_, 1.5 mM ATP, 0.2 mg/ml bovine serum albumin, 0.5 U/ml yeast inorganic pyrophosphatase and a proper enzyme dilution. Assays were initiated by addition of Glc-1P at a final concentration of 1.5 mM in a total volume of 50 μl. The reaction mixture was incubated for 10 min at 37°C and terminated by adding the Malachite Green reactive. The complex formed with the released P_i_ was measured at 630 nm.

Alternatively, activity was measured by the radiometric coupled assay method (Yep et al., 2004), measuring the synthesis of ADP-[^14^C]Glc from [^14^C]Glc-1P and ATP. The standard reaction mixture contained 100 mM MOPS buffer (pH 8.0), 10 mM MgCl_2_, 1 mM [^14^C]Glc-1P (100–1000 cpm/nmol), 1.5 mM ATP, 0.5 units/ml inorganic pyrophosphatase, and 0.2 mg/ml BSA, plus enzyme in a total volume of 0.2 ml. Reaction mixtures were incubated for 10 min at 37°C and terminated by heating in a boiling-water bath for 1 min. The ADP[^14^C]Glc was then converted to [^14^C]glycogen by the addition of *E. coli* GSase and non-radioactive glycogen as a primer. Glycogen formed was precipitated and washed, and the radioactivity measured in a scintillation counter.

One unit of activity (U) is defined as the amount of enzyme catalyzing the formation of 1 μmol of product per min, under conditions above described in each case.

### Calculation of Kinetic Constants

Saturation curves were performed by assaying enzyme activity at different concentrations of the variable substrate or effector and saturating levels of the others. Experimental data were plotted as enzyme activity (U/mg) *versus* substrate (or effector) concentration (mM), and kinetic constants were determined by fitting the data to the Hill equation as described elsewhere (Ballicora et al., 2007). Fitting was performed with the Levenberg–Marquardt non-linear least-squares algorithm provided by the computer program Origin^TM^. Hill plots were used to calculate the Hill coefficient (*n*_H_), the maximal velocity (*V*_max_), and the kinetic constants that correspond to the activator, substrate, or inhibitor concentrations giving 50% of the maximal activation (*A*_0.5_), velocity (*S*_0.5_), or inhibition (*I*_0.5_). All kinetic constants are the mean of at least three independent sets of data, which were reproducible within ±10%.

## Results

### Recombinant Expression and Purification of ADP-Glc PPase From *R. jostii* RHA1

**FIGURE 1:**
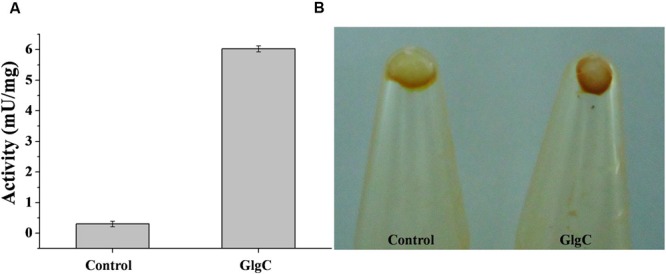
**Heterologous expression of *Rhodococcus jostii* ADP-Glc PPase in *Escherichia coli* strain AC70R1-504. (A)** Specific activity in soluble fractions of crude extract from cells transformed with pMAB5 without *RjoglgC* insert (control) or cells transformed with [pMAB5/*RjoglgC*] to express *R. jostii* ADP-Glc PPase (GlgC). **(B)** Iodine staining of pellets cell from *E. coli* AC70RI-504 control and after expression of *R. jostii* ADP-Glc PPase.

We identified the *glgC* gene (ID 4223526, *RjoglgC*), coding for ADP-Glc PPase in the genome of *R. jostii* RHA1. Then, specific oligonucleotides primers were designed for *glgC* amplification using established PCR procedures and identity of the amplified gene was confirmed by its complete sequencing. The *RjoglgC* gene (1,215 bp) encodes a protein with a theoretical molecular mass of 43.85 kDa having high amino acidic sequence identity with ADP-Glc PPases from other actinobacteria already characterized: 65.7, 71.2, and 83.0% regarding the enzymes from *Streptomyces coelicolor* (Asencion Diez et al., 2012), *Corynebacterium glutamicum* (Seibold et al., 2007), and *M. tuberculosis* (Asencion Diez et al., 2015), respectively. The *RjoglgC* gene was cloned into pMAB5 and pET28c plasmids for different experimental purposes. The construct [pMAB5/*RjoglgC*] was developed to complement an *E. coli* strain with low ADP-Glc PPase activity, as reported before (Iglesias et al., 1993; Asencion Diez et al., 2013a). As shown in **Figure 1**, crude extracts from *E. coli* AC70RI-504 cells transformed with [pMAB5/*RjoglgC*] exhibited ADP-Glc PPase activity values (6.0 ± 0.1 mU/mg) 20-fold higher than those from untransformed (or transformed with pMAB5 without *RjoglgC* insert) control cells. In addition, we observed that *E. coli* AC70RI-504 harboring the recombinant *RjoglgC* gene accumulated higher amounts of glycogen when compared with control cells (**Figure 1**); which suggests that the heterologous expression renders a functional *R. jostii* ADP-Glc PPase.

**FIGURE 2:**
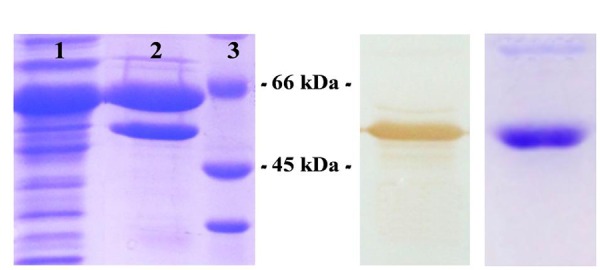
**Co-expression of *R. jostii* ADP-Glc PPase with GroES-GroEL chaperone system. (A)** SDS-PAGE of samples corresponding to the soluble fraction of crude extract from co-transformed *E. coli* cells (lane 1), and the latter after capture with Ni-IDA resin (lane 2), Molecular mass markers (lane 3). **(B)** Immunodetection of Western blotted lane 2 of **(A)** using antibodies raised against *Mycobacterium tuberculosis* ADP-Glc PPase. **(C)** SDS-PAGE of the purified sample eluted after a second round of IMAC performed with Co-IDA.

The construct [pET28c/*RjoglgC*] was designed to achieve high expression levels of *R. jostii* ADP-Glc PPase fused to an N-term His-tag for easy purification. The use of this plasmid construct enabled the generation of high amounts of the recombinant enzyme mainly as an insoluble protein (no matter the culture conditions or the host cells employed, see Supplementary Figure S1), with no detectable activity in any of the expression options assayed. Soluble (and active) *R. jostii* ADP-Glc PPase was obtained with a co-expression strategy using the GroEL/GroES chaperones system (Supplementary Figure S2), a procedure previously reported for the production of the orthologous enzyme from *S. coelicolor* (Asencion Diez et al., 2012). The enzyme in the soluble fraction was partially purified with Ni-IDA (**Figure 2A**), which rendered a sample enriched in the chaperones and ADP-Glc PPase. The latter was immunodetected using antibodies raised against the homologous *M. tuberculosis* enzyme, as shown **Figure 2B** and Supplementary Figure S3. A new round of IMAC using Co instead of Ni for protein elution allowed to obtain a highly purified *R. jostii* ADP-Glc PPase (**Figure 2C**) with more than 50-fold increase (from the crude extract) in its specific activity to reach a value of 0.3 U/mg. Under these conditions the purified recombinant enzyme resulted unstable, as activity decreased by ∼50% in 24 h (not shown). Afterward, different storage conditions were analyzed for activity stabilization. It was found that concentration and dialysis to a buffer containing 50 mM HEPES pH 8.0, 0.5 mM DTT, 0.1 mM EDTA, 20% w/v sucrose, followed by storage at -80°C rendered an enzyme stable for at least 12 months. The purified enzyme eluted as a protein of 190 kDa when analyzed by Superdex 200 size exclusion chromatography (data not shown), thus indicating a homotetrameric structure for the functional *R. jostii* ADP-Glc PPase.

### Characterization of the Kinetic and Regulatory Properties of *R. jostii* ADP-Glc PPase

**FIGURE 3:**
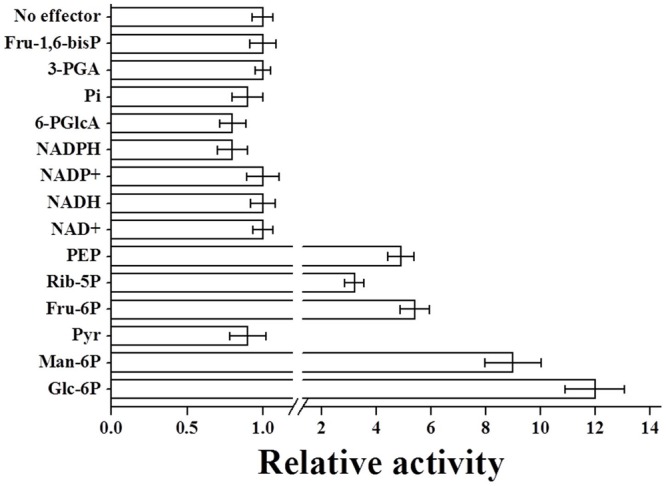
**Relative activity of *R. jostii* ADP-Glc PPase in presence of different metabolites.** The relative activities were calculated as the ratio between the enzyme activity in presence of the effector and the activity in its absence. The value of 1 correspond to the *R. jostii* ADP-Glc PPase *V*_max_ (0.39 ± 0.01 U/mg). The respective metabolite concentration was 2 mM in all cases.

The purified *R. jostii* ADP-Glc PPase was characterized in the physiological ADP-Glc synthesis direction using 5 mM MgCl_2_, since Mg^2+^ is the typical essential cofactor for this enzyme family (Ballicora et al., 2003, 2004). The *R. jostii* ADP-Glc PPase exhibited saturation kinetics slightly deviated from hyperbolic behavior for ATP (*n*_H_ = 1.2) and Glc-1P (*n*_H_ = 0.9), with substrates *S*_0.5_ values around 1–2 mM and a *V*_max_ of 0.39 U/mg (see Supplementary Figure S4). We also performed activation-inhibition assays to explore if the recombinant *R. jostii* enzyme is affected by molecules known to modulate ADP-Glc PPase activity in other bacteria (Ballicora et al., 2003, 2004; Asencion Diez et al., 2012). Results in **Figure 3** indicate that the *R. jostii* ADP-Glc PPase behavior is mainly activated by Glc-6P, Man-6P, Fru-6P, Rib-5P, and PEP; while inhibited by Pyr, NADPH, and 6-PGlcA.

The kinetic behavior of *R. jostii* ADP-Glc PPase activators was further analyzed, as shown in Supplementary Figure S5. Curves for each one were conducted (see Supplementary data sheet) to calculate maximal activation degrees and *A*_0.5_ values, as detailed in **Table 1**. Saturation kinetics indicated that all the five activators enhanced the enzyme activity to a similar extent, increasing 8- and 13-fold although with different *A*_0.5_ values. When ADP-Glc PPase is activated by many effectors, they show marked differences in the degree of activation (Ballicora et al., 2003, 2004) as in the case of the enzyme from *S. coelicolor* (Asencion Diez et al., 2012). Herein, we show that both Glc-6P and Man-6P activate the *R. jostii* ADP-Glc PPase at sub-millimolar *A*_0.5_ higher values (both metabolites exhibiting the highest apparent affinities) and showed a marked sigmoidal behavior. Conversely, affinity toward Fru-6P and Rib-5P was found to be in the millimolar range, while that for PEP was in the mid-range, with the lowest cooperative behavior (**Table 1**).

**Table 1:** Kinetic parameters for allosteric effectors of *Rhodococcus jostii* ADP-Glc PPase.

Effector	*A*_0.5_ (mM)	*n*_H_	Activation^a^ (-fold)
Glc-6P	0.088 ± 0.005	2.2 ± 0.2	12
Man-6P	0.056 ± 0.005	2.9 ± 0.1	9
Fru-6P	2.2 ± 0.1	1.7 ± 0.1	13
Rib-5P	4.0 ± 0.5	1.80 ± 0.08	8
PEP	0.3 ± 0.2	1.20 ± 0.05	9

*^a^Activation was calculated as the ratio of *V*_max_ determined from the effector saturation curve over the *V*_max_ of the enzyme in the absence of effector.*

**FIGURE 4:**
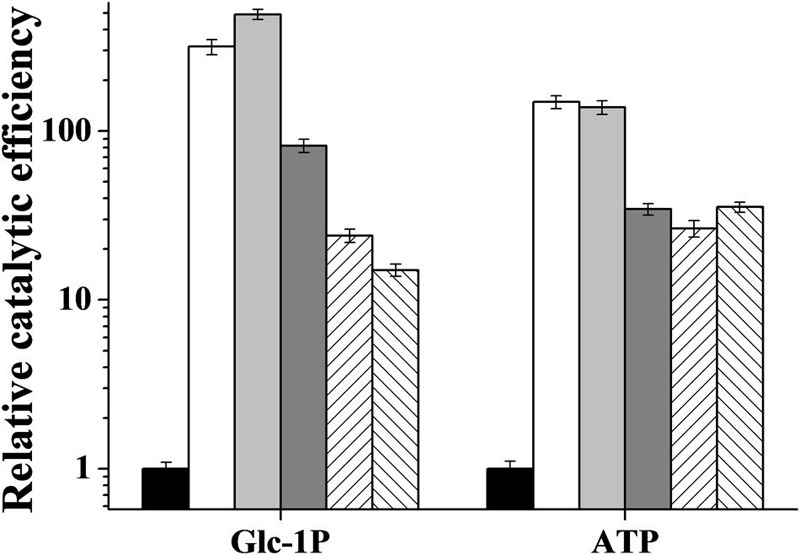
**Relative catalytic efficiency of *R. jostii* ADP-Glc PPase in absence (black bars) or in presence of 0.5 mM Glc-6P (white bars), 0.5 mM Man-6P (light gray bars), 5 mM Fru-6P (dark gray bars), 5 mM Rib-5P (right diagonal bars) or 5 mM PEP (left diagonal bars)**.

The *R. jostii* ADP-Glc PPase allosteric activators modified both *V*_max_ and substrates *S*_0.5_ values. As shown in **Table 2**, at saturating concentrations, activators increased the enzyme’s relative affinity toward both substrates, at a higher extent for Glc-1P (between 5- and 25-fold) than for ATP (between 2- and 8-fold). Considering the combined effects, the enzyme catalytic efficiency [ratio *V*_max_/*S*_0.5_; equivalent to *k*_cat_/*K*_m_ for hyperbolic kinetics (Cornish-Bowden and Cardenas, 2010)] was significantly affected by the activators, as illustrated in **Figure 4**. Noteworthy, the main activation effect of Man-6P and Glc-6P consists of an increase of two orders of magnitude (note the logarithmic scale in **Figure 4**) in catalytic efficiency for both substrates (about 300- and 100-fold increase for Glc-1P and ATP, respectively). Also important are the activation effects between 20- and 80-fold induced by Fru-6P, Rib-5P or PEP, when present at saturating levels in the assay medium.

**Table 2:** Kinetic parameters of *R. jostii* ADP-Glc PPase determined in the absence and in the presence of activators.

	Glc-1P		ATP		
	*S*_0.5_ (mM)	*n*_H_	*S*_0.5_ (mM)	*n*_H_	*V*_max_
Control	1.8 ± 0.2	0.90 ± 0.08	1.2 ± 0.1	1.2 ± 0.1	0.39 ± 0.02
Glc-6P (0.5 mM)	0.085 ± 0.008	1.1 ± 0.1	0.238 ± 0.009	1.6 ± 0.2	4.80 ± 0.08
Man-6P (0.5 mM)	0.071 ± 0.006	1.30 ± 0.09	0.145 ± 0.008	1.30 ± 0.08	3.5 ± 0.1
Fru-6P (5 mM)	0.16 ± 0.05	0.7 ± 0.1	0.5 ± 0.1	1.10 ± 0.09	5.1 ± 0.1
Rib-5P (5 mM)	0.4 ± 0.3	0.60 ± 0.04	0.47 ± 0.09	1.3 ± 0.1	3.12 ± 0.02
PEP (5 mM)	0.17 ± 0.05	0.8	0.43 ± 0.07	1.4 ± 0.1	3.47 ± 0.01

**FIGURE 5:**
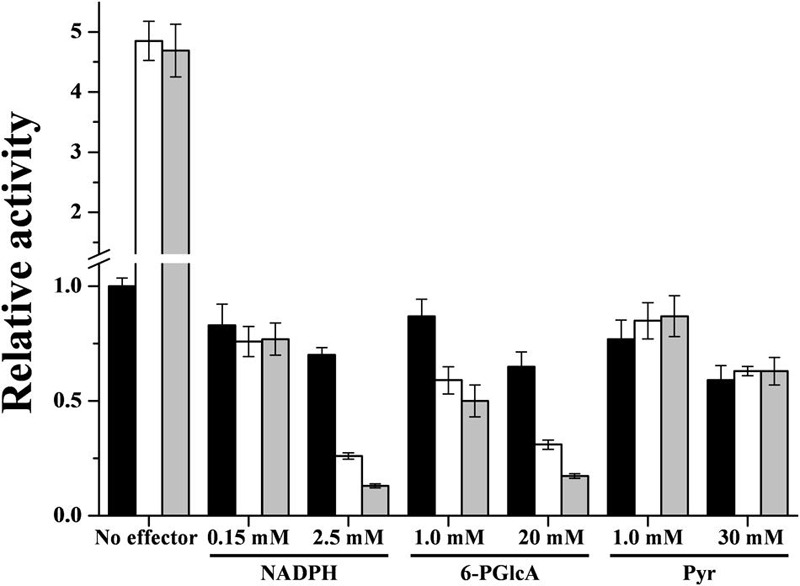
**Interplay between effectors of the *R. jostii* ADP-Glc PPase.** Interaction of Glc-6P or Man-6P with NADPH, 6-PGlcA and Pyr. Assays were performed in absence of any activator (black bars) or in presence of 0.1 mM Glc-6P (white bars) or 0.05 mM Man-6P (gray bars). The value of 1 represents the activity of the enzyme in absence of effector (0.39 ± 0.01 U/mg).

Regarding *R. jostii* ADP-Glc PPase inhibitors (NADPH, 6-PGlcA and Pyr, see **Figure 3**), they exhibited a partial effect, inhibiting the enzyme activity by only 50% or less (see Supplementary Figure S5). Enzyme inhibition was affected by the presence of the activators Glc-6P or Man-6P. Sub-saturating levels of each activator (around the respective *A*_0.5_ value) produced a marked increase in the degree of inhibition to 20–30% remnant activity at the higher NADPH or 6-PGlcA concentration (**Figure 5**). On the other hand, the inhibitory effect of Pyr was not affected by the presence of the activators. Together, the *in vitro* results obtained suggest that the *in vivo* activity of ADP-Glc PPase from *R. jostii* may be critically determined by the relative concentrations of main activators and inhibitors.

### *R. jostii* ADP-Glc PPase Uses Alternative Substrates

**FIGURE 6:**
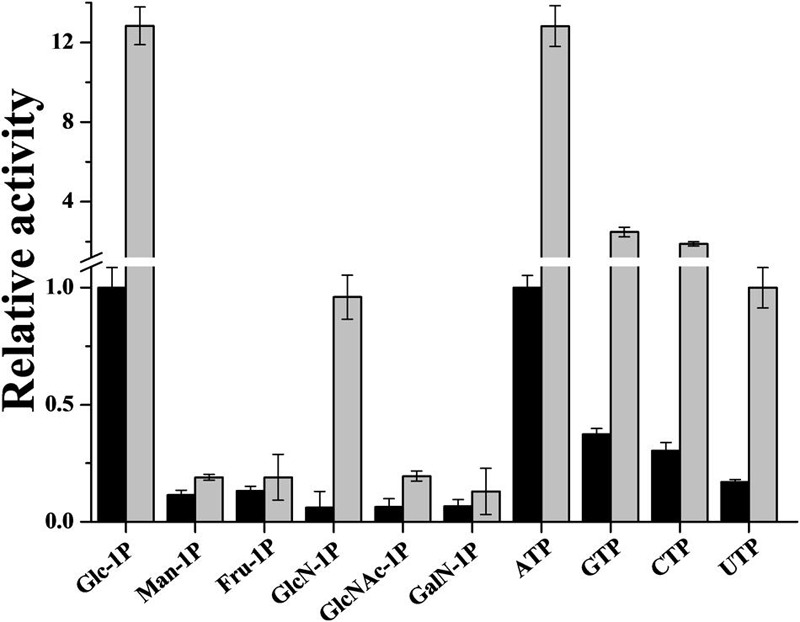
***R. jostii* ADP-Glc PPase promiscuity for alternatives sugar-1P and NTP.** Assays were performed in absence (black bars) or in presence of 0.5 mM Glc-6P (gray bars). The value of 1 correspond to the *R. jostii* ADP-Glc PPase *V*_max_ (0.39 ± 0.01 U/mg). The alternative substrate concentration was 2 mM in all cases.

The *R. jostii* ADP-Glc PPase was also analyzed regarding its ability to use alternative substrates. The enzyme is active when ATP and Glc-1P are replaced by corresponding concentrations of NTPs and sugar-1Ps, respectively (**Figure 6**). GTP and CTP showed 40–35% activity compared to ATP (**Figure 6**). On the other hand, *R. jostii* ADP-Glc PPase used alternative sugar-1Ps reaching activity values up to 12% compared to Glc-1P (**Figure 6**). The enzyme’s promiscuity (lack of specificity) for the use of alternative substrates was analyzed in presence of a saturating concentration of Glc-6P, the common activator in actinobacterial ADP-Glc PPases. Regarding NTPs, Glc-6P induced increments of 9.3-, 9.5-, or 6.0-fold in activity assayed with GTP, CTP, or UTP, respectively (**Figure 6**); although the hexose-P exhibited no effect on the enzyme’s apparent affinity toward the alternative substrates (not shown). Glc-6P also affected the use of sugar-1Ps, increasing the enzyme activity 1.5–3 times, with the exception of GlcN-1P that induced an order of magnitude increase (**Figure 6**). Actually, in the absence of activator the saturation curves showed no clear plateau up to 10 mM GlcN-1P (*S*_0.5_ > 6 mM), however in the presence of 0.5 mM Glc-6P a slightly sigmoidal (*n*_H_ = 1.5) saturation behavior was observed, with an *S*_0.5_ value of 3.3 mM (Supplementary Figure S6).

## Discussion

Herein, we show the kinetic and regulatory characterization of the ADP-Glc PPase from *R. jostii*. Our results contribute to a better understanding about glycogen synthesis in this oleaginous bacterium and a plausible role as a carbon capacitor interconnecting growth and lipid biosynthesis.

### Kinetic and Regulatory Properties of the *R. jostii* ADP-Glc PPase

Kinetic characteristics of enzymes related to glycogen synthesis in Gram-positive organisms are less well-known than those from Gram-negative prokaryotes. Recently, specific properties regarding regulation of the classical glycogen synthesis pathway in Gram-positive bacteria were identified (Asencion Diez et al., 2012, 2013a, 2015). Glycogen synthesis via an alternative pathway using maltose-1P as precursor was recently described in Actinobacteria. This is known as the GlgE pathway [because of the key enzyme GlgE (EC 2.4.99.16)] and implies that glycogen and trehalose [α-D-glucopyranosyl-(1→1)-α-D-glucopyranoside] metabolisms are interrelated (Elbein et al., 2010; Chandra et al., 2011), operating as an alternative to the classical GlgCA route that is finely regulated by metabolites at the level of ADP-Glc synthesis (Ballicora et al., 2003, 2004). The analysis of the genome from *R. jostii* RHA1 shows the presence of a single *glgC* gene, albeit the organism presents many gene duplications, such as genes coding for UDP-Glc PPase (EC 2.7.7.9) or trehalose-6P synthase (McLeod et al., 2006; Tischler et al., 2013). Herein, we characterized the ADP-Glc PPase from *R. jostii* to further understand the kinetic and regulatory properties of an actinobacterial enzyme catalyzing a key step in glucan biosynthesis.

The ADP-Glc PPase from *R. jostii* was characterized as a homotetrameric protein exhibiting similar *S*_0.5_ values for substrates to those calculated for the enzyme from *M. tuberculosis*, but fivefold higher than those from the *S. coelicolor* enzyme, although with levels of activity in the same order of magnitude than the latter. Comparatively, the catalytic efficiency of the ADP-Glc PPase from *R. jostii* exhibits the lowest values with respect to homologous proteins from *S. coelicolor* and *M. tuberculosis* or even other PPases so far characterized (Iglesias et al., 1991; Charng et al., 1992; Ballicora et al., 1995, 2002; Ugalde et al., 1998; Uttaro et al., 1998; Gomez-Casati and Iglesias, 2002; Bosco et al., 2009; Asencion Diez et al., 2012, 2013a,b, 2014, 2015; Machtey et al., 2012; Ebrecht et al., 2015a,b). Thus, the *R. jostii* ADP-Glc PPase might be an enzyme with low basal activity, as proposed for the ADP-Glc PPase from *Streptococcus mutans* with homotetrameric conformation (GlgC, with similar values of catalytic efficiency; Asencion Diez et al., 2013a). Instead, the activity of the *R. jostii* enzyme strongly depends on the presence of metabolites acting as allosteric regulators (as detailed below). The kinetic behavior agrees with the biological properties of an organism with slow duplication time, storing lipids rather than glycogen (Hernandez et al., 2008; Hernandez and Alvarez, 2010). Indeed, *R jostii* accumulates glycogen at levels equivalent to 2% of cell dry weight (CDW; Hernandez et al., 2008), unlike in *E. coli* where glycogen attain equivalent levels to 50% of CDW (Fung et al., 2013).

The specificity toward allosteric regulators by *R. jostii* ADP-Glc PPase shows similarities with the enzyme from other Gram-positive bacteria, the non-firmicutes *S. coelicolor* and *M. tuberculosis* (Asencion Diez et al., 2012, 2015). Characteristically, Glc-6P is characteristically a key activator of actinobacteria ADP-Glc PPases; whereas NADPH is a critical inhibitor of the enzyme in *R. jostii* and *S. coelicolor* (Asencion Diez et al., 2012). Besides, Fru-6P, Man-6P, Rib-5P, and PEP would play an important role in determining levels of activity in the pathway of glycogen synthesis in rhodococci. An important feature is the cross-talk between effectors to regulate *R. jostii* ADP-Glc PPase, as the enzyme activity would be finely modulated according the levels of intermediaries of the different metabolic pathways operating in the microorganism under different growing conditions. This picture is in agreement with the idea that ADP-Glc PPase is the key regulatory enzyme in the glycogen synthesis pathway in all prokaryotes including cyanobacteria (Ballicora et al., 2003, 2004).

In general, ADP-Glc PPases exhibit a high degree of specificity for its substrates (Ballicora et al., 2003, 2004; Preiss, 2009). We previously reported that both *S. coelicolor* and *M. tuberculosis* ADP-Glc PPases are highly specific for ATP (Asencion Diez et al., 2012, 2013a, 2015), contrasting with results showed in this work regarding the *R. jostii* ADP-Glc PPase promiscuity toward GTP and CTP. To the best of our knowledge, this is the first time that lack of substrate specificity toward NTPs is shown in an ADP-Glc PPase from bacteria (Ballicora et al., 2003), including actinobacteria (Asencion Diez et al., 2012, 2015). The closest example is the enzyme from *Nitrosomonas europaea*, although the latter showed less than 10% activity with other NTPs and its activity with GTP was almost no detectable (Machtey et al., 2012). Additionally, we report herein, for the first time, an unusual activity regarding the high preference for GlcN-1P. In a previous work Bejar et al. (2006) studied the structure of the Glc-1P binding region, and identified specific amino acids that interact with the substrate. The *R. jostii* ADP-Glc PPase has all these homolog residues (Glu^194^, Ser^212^, Tyr^216^, Asp^239^, Trp^274^, and Asp^276^), but Phe^240^ is replaced by a Met. Since the role of Phe^240^ might be merely structural affecting the Glc-1P apparent affinity (see Bejar et al., 2006), little could be ascribed to this mutation as responsible of the sugar-1P promiscuity in the *R. jostii* enzyme. We also show that Glc-6P increases the apparent affinity of GlcN-1P. Current work in our laboratory is devoted to further characterize the apparent lack of substrate specificity (promiscuity) by the ADP-Glc PPase from *R. jostii*, as well as the distinctive activation by Glc-6P, to further understand evolutionary aspects of this enzyme family.

### Allosteric Regulation of the ADP-Glc PPase and Its Role in the Physiological Behavior of *R. jostii*

It is well-established that ADP-Glc PPases are regulated by metabolites from the major carbon utilization routes in different organisms, and have been classified with respect to their effector specificity (Ballicora et al., 2003, 2004; Preiss, 2009). We propose a new component to be added to this classification, i.e., the actinobacterial ADP-Glc PPases activated by Glc-6P. However, although the *R. jostii, S. coelicolor*, and *M. tuberculosis* ADP-Glc PPases exhibit a high degree of identity, they possess different effector sensitivity and level of response [this work and (Asencion Diez et al., 2012, 2013a, 2015)]. Results in this study indicate that *R. jostii* ADP-Glc PPase activity depends on its interaction with eight different metabolites from the glycolytic pathways. Indeed, the relative concentration of each of these effectors and their combined action will determine the overall enzyme activity (and consequently the impact on this polyglucan synthesis).

Based on recent integrated proteomic study, we performed a predictive analysis for a comprehensive view on the ADP-Glc PPase role and, consequently, glycogen metabolism in the *R. jostii* physiology. Strain RHA1 of *R. jostii* is able to produce significant amounts of TAG (approximately 55–60% of CDW) and minor amounts of glycogen (∼2–3% of CDW) during cultivation on gluconate. Cells accumulate glycogen mainly during exponential growth phase while its content decreases during the stationary phase in which massive TAG biosynthesis and accumulation occur (Hernandez et al., 2008). Proteomic studies in this oleaginous bacterium comparing protein abundances during cultivation in media with and without nitrogen source, using gluconate as sole carbon source in both conditions. The presence of nitrogen promoted cell growth whereas its absence elicited lipid accumulation (Davila Costa et al., 2015).

**FIGURE 7:**
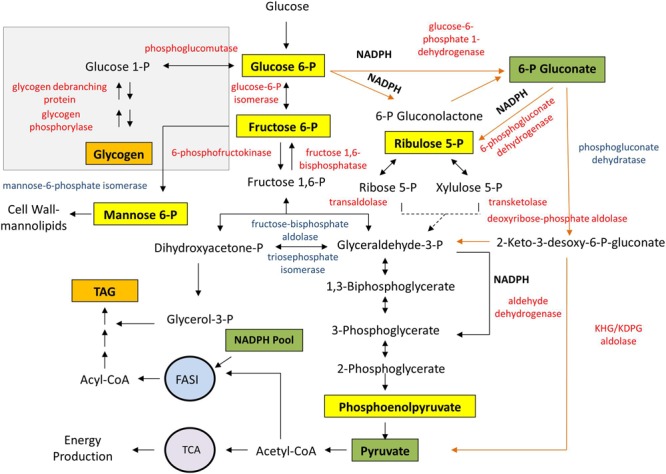
**Glycogen metabolism integrated to glycolytic pathways and TAG biosynthesis in the oleaginous *R. jostii* RHA1.** Proteins in red showed significantly high abundance under TAG-accumulating conditions, whereas proteins in blue exhibited high abundance in both, growth and TAG-accumulating conditions, in the label-free proteome (Davila Costa et al., 2015). Orange arrows indicate the ED pathway reactions. Yellow boxes denote activator metabolites while green ones indicate inhibitory metabolites of the ADP-Glc PPase from *R. jostii* RHA1.

**Figure 7** shows the enzyme pattern and the landscape of metabolites from glycolysis as they happen in strain RHA1 during accumulation of TAG. The abundance of enzymes involved in glycogen degradation, such as glycogen phosphorylase (GlgP, EC 2.4.1.1), glycogen debranching enzyme (GlgX, EC 3.2.1.33) and phosphoglucomutase (PGM, EC 5.4.2.2), was significantly increased during cell cultivation under conditions leading to TAG accumulation (**Figure 7**). Oppositely, no changes were detected in the abundance of enzymes involved in glycogen synthesis, including the ADP-Glc PPase, when comparing proteomic studies with cells cultivated with and without nitrogen source (Davila Costa et al., 2015). Proteins from the classical glycolytic Embden-Meyerhoff (EMP) route were present in cells cultured with or without nitrogen source (**Figure 7**). Glycolytic enzymes Glc-6P isomerase (PGI, EC 5.3.1.9) and 6-phosphofructokinase (PFK, EC 2.7.1.11) as well as the gluconeogenic enzyme fructose-1,6-biphosphatase (FBPase, EC 3.1.3.11) were also increased in strain RHA1 during lipogenesis leading to TAG accumulation.

Several enzymes from the Entner-Doudoroff (ED) and pentose-phosphate (PPP) pathways were highly expressed in strain RHA1 under nitrogen-limiting TAG-accumulating conditions (**Figure 7**). Both pathways generate reducing equivalents in the form of NADPH, in contrast to EMP which produces NADH. NADPH is required as cofactor for the biosynthesis of fatty acids, which are main precursors for TAG production. In addition, three putative NADP-dependent glyceraldehyde 3-P dehydrogenase enzymes (EC 1.2.1.9), which oxidize glyceraldehyde-3-P with the generation of NADPH, showed a high abundance during TAG accumulation (**Figure 7**).

The enzyme Man-6P isomerase (EC 5.3.1.8), which interconverts Man-6P and Fru-6P, was present and similarly abundant under both conditions analyzed (**Figure 7**). Man-6P is probably a key intermediate for the synthesis of diverse cell wall-mannolipids in rhodococci, such as lipoarabinomannan, among others. Since Glc-6P, Man-6P, and Fru-6P are *R. jostii* ADP-Glc PPase activators, Man-6P isomerase might be involved in regulating hexose-6P relative concentrations together with PGM, PGI, PFK, and FBPase.

The available proteomic information for the oleaginous *R. jostii* RHA1 indicates that cells require a substantial metabolic remodeling likely involving 100s of individual reactions, when cells shift from a growth- to a TAG-accumulating state. The variation in the enzyme’s expression patterns from the glycolytic routes during TAG accumulation may reflect the dynamics of the different ADP-Glc PPase effectors in the metabolic scenario of the oleaginous strain RHA1. During lipogenesis, the major function of glycolysis in RHA1 is to provide carbons from phosphorylated sugars for the *de novo* lipid synthesis. In this context, the metabolic network has to supply a high carbon flux toward lipogenic routes, and a sufficient supply of reducing equivalents for the massive generation of fatty acids. Apparently, during lipid accumulation, the oleaginous strain RHA1 exhibits several metabolic changes, including activation of ED and PPP pathways, which generate the required reducing equivalents in the form of NADPH. Although, the ED pathway is less efficient than EMP for energy generation, the former is advantageous because requires fewer enzymes and avoids the loss of carbon, which can be conserved for lipogenesis. The observed ADP-Glc PPase promiscuity for NTP may enable adaptation of its activity to this low energy cellular environment.

According to our studies, one of the main differences of *R. jostii* RHA1 ADP-Glc PPase compared to those from related actinobacteria, is the inhibitory effect of 6-PGlcA on the enzyme activity, which is a key metabolite of ED pathway. The activation of this glycolytic route during lipid accumulation, together with the formation of abundant NADPH by different reactions, such as those of the PPP, ED and NADP-dependent glyceraldehyde-3-P dehydrogenase enzymes, promote a decrease of the ADP-Glc PPase activity within the metabolic network of RHA1 (see **Figure 7**). Moreover, the availability of Glc-6P together with high levels of NADPH may increase the inhibition of the ADP-Glc PPase enzyme. These changes in ADP-Glc PPase activity should impact glycogen metabolism explaining its accumulation as well as its decrease during the exponential or stationary growth phases, respectively, as reported in *R. jostii* RHA1 and other rhodococci (Hernandez et al., 2008; Hernandez and Alvarez, 2010). On the other hand, the enzymes involved in glycogen degradation are transcriptionally activated in order to provide Glc-6P for glycolytic pathways.

The significant abundance of enzymes from gluconeogenesis, such as PEP carboxykinase (EC 4.1.1.32) and FBPase, together with PFK from EMP pathway, may suggest that part of the downstream metabolites (i.e., from the tricarboxylic acid cycle) can be redirected toward gluconeogenesis for Glc-6P regeneration, which is then partitioned into the ED and PPP routes. This metabolic configuration may confer the following advantages for RHA1 cells: (1) the dynamics of the glycolytic and gluconeogenic pathways running simultaneously may generate a cycle, which can increase the efficiency for acetyl-CoA and NADPH production; and (2) the redirection of Glc-6P to ED pathway may avoid the reciprocal regulation of the EMP and gluconeogenesis pathways, which frequently occur in actinobacteria (Hollinshead et al., 2015). Here, it is worth mentioning that in *M. tuberculosis*, an actinobacterium closely related to rhodococci (McGuire et al., 2012), it has been reported the relevance of the gluconeogenic pathway for virulence/pathogenesis (Puckett et al., 2014; Ganapathy et al., 2015) and that it can happens concomitantly with EMP (Marrero et al., 2010; Rhee et al., 2011).

The metabolic scenario and the availability of glycolytic intermediates seem to be finely regulated in the metabolic network of the oleaginous *R. opacus* PD630 (Hollinshead et al., 2015), as probably occurs in *R. jostii* RHA1. In this context, glycogen may be part of a sensing mechanism for dealing with sugar excess in the cellular metabolism. Thus, glycogen synthesis may act to attenuate phosphorylated sugar fluxes through the glycolytic pathways for ensuring adequate supply of energy, reducing equivalents, and precursors according to cellular demand. The modulation of glycogen synthesis and accumulation in RHA1 cells occurs allosterically through the control of the ADP-Glc PPase activity, as shown herein. Moreover, the activity of ADP-Glc PPase might be also integrated with cell growth and division processes since the enzyme is activated by Man-6P. An excess of Man-6P could induce the use of Glc-6P for glycogen synthesis as a way to control flux while keeping sugar metabolism in balance during cell growth. However, the relationship between ADP-Glc PPase and growth/division in RHA1 needs further investigation.

Taken together, the results presented in this study indicate that glycogen may not play the role of an endogenous carbon and energy source in rhodococci but might rather buffer and control the flux of phosphorylated sugars through the glycolytic pathways and central metabolism (see **Figure 7**). The allosteric modulation of the ADP-Glc PPase activity may be part of a sensitive and evolutionarily conserved mechanism for oleaginous rhodococci to efficiently conserve carbon and energy during growth and likely division.

## Author Contributions

Conceived and designed the experiments: AI and HA. Performed the experiments: AC, MA, and JD. Analyzed the data: AC, MA, AI, and HA. Contributed reagents/materials/analysis tools: AI, MA, and HA. Wrote the paper: AC, MA, AI, and HA.

## Conflict of Interest Statement

The authors declare that the research was conducted in the absence of any commercial or financial relationships that could be construed as a potential conflict of interest.

## Footnotes

1 http://www.ncbi.nlm.nih.gov/nuccore/111017022
