# Supplementary material for: On the Kinetic and Allosteric Regulatory Properties of the ADP-Glucose Pyrophosphorylase from Rhodococcus jostii: An Approach to Evaluate Glycogen Metabolism in Oleaginous Bacteria
Source: Front Microbiol. 2016 Jun 2;7:830. doi: 10.3389/fmicb.2016.00830 (PMC4890535; doi:10.3389/fmicb.2016.00830)
Supplement: Supplementary file 2 [file Presentation_1.PDF]

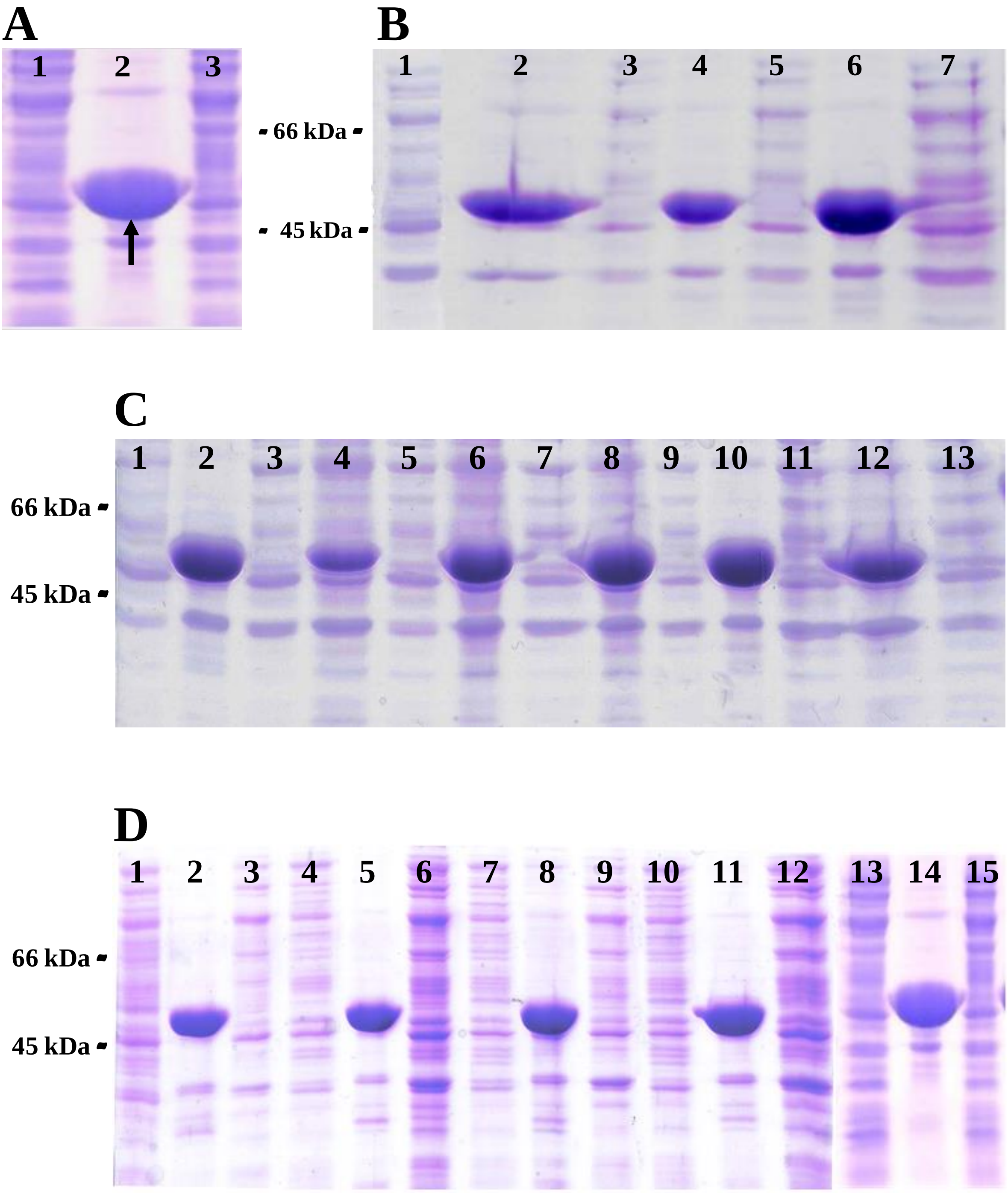

Supplemental Figure 1: SDS-PAGE analysis of *R. jostii* ADP-Glc PPase expression using the vector pET28c. All expression assays were carried out at 16 °C during 16 h with 0.1 mM IPTG, unless otherwise specified. **A:** Expression analysis in *E. coli* BL21 (DE3) cells. Soluble fraction of cells transformed with [pET28c] without gene inserted (control) **(1)**, insoluble **(2)** and soluble **(3)** fraction of

cells containing [pET28c/*RjoglgC*] construction. **B:** ADP-Glc PPase expression using *E. coli* BL21 (DE3) cells transformed with [pET28c/*RjoglgC*] and grown in different culture media. Soluble fraction of control cells **(1)**, insoluble **(2)** and soluble **(3)** fraction of cells grown in LB medium; insoluble **(4)** and soluble **(5)** fraction in LB-Glc (1.5% w/v) medium; insoluble **(6)** and soluble **(7)** fraction in YT2X medium. **C:** Expression assays with *E. coli* BL21 (DE3) cells using different induction conditions. Soluble fraction of control cells **(1)**, insoluble **(2)** and soluble **(3)** fraction of cells induced with 0.2 mM IPTG during 4 h at 37 °C; insoluble **(4)** and soluble **(5)** fraction of cells induced with 0.2 mM IPTG, 4 h, 16 °C; insoluble **(6)** and soluble **(7)** fraction of cells induced with lactose 2.5 g/l during 16 h at 16 °C; insoluble **(8)** and soluble **(9)** fraction of cells induced with 0.3 mM IPTG, 16 h at 16 °C; insoluble **(10)** and soluble **(11)** fraction of cells induced with 0.1 mM IPTG during 16 h at 20 °C; insoluble **(12)** and soluble **(13)** fraction of cells induced with 0.1 mM IPTG, 16 h, 37 °C. **D:** Expression assays using [pET28c/*RjoglgC*] construction in different *E. coli* strains. Soluble fraction of control *E. coli* Codon plus **(1)**, insoluble **(2)** and soluble **(3)** fraction of this cells transformed with the above construction; soluble fraction of control *E. coli* B834 **(4)**, insoluble **(5)** and soluble **(6)** fraction of the same strain transformed; soluble fraction of control *E. coli* Rossetta **(7)**, insoluble **(8)** and soluble **(9)** fraction of this strain transformed; soluble fraction of control *E. coli* Tunner **(10)**, insoluble **(11)** and soluble **(12)** fraction if this cells containing the construction; soluble fraction of control *E. coli* SHuffle **(13)**, insoluble **(14)** and soluble **(15)** fraction of this strain transformed.

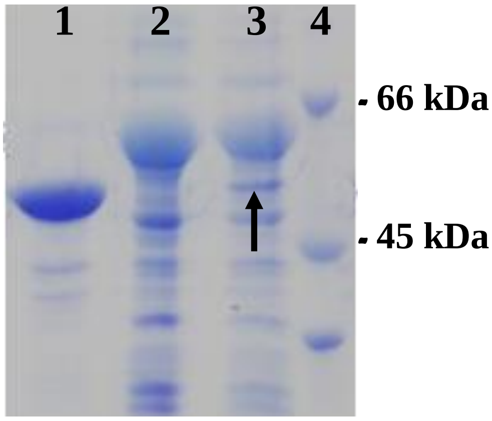

<u>Supplemental Figure 2</u>: Co-expression of *R. jostii* ADP-Glc PPase with GroES-GroEL chaperone system. SDS-PAGE of crude extract samples corresponding to the insoluble fraction from co-transformed cells (lane 1), the soluble fraction of control cells transformed with chaperone system (lane 2), and the soluble fraction of co-transformed cells (lane 3) [the arrow indicates the soluble expression of *R. jostii* ADP-Glc PPase]. Molecular mass markers (lane 4).

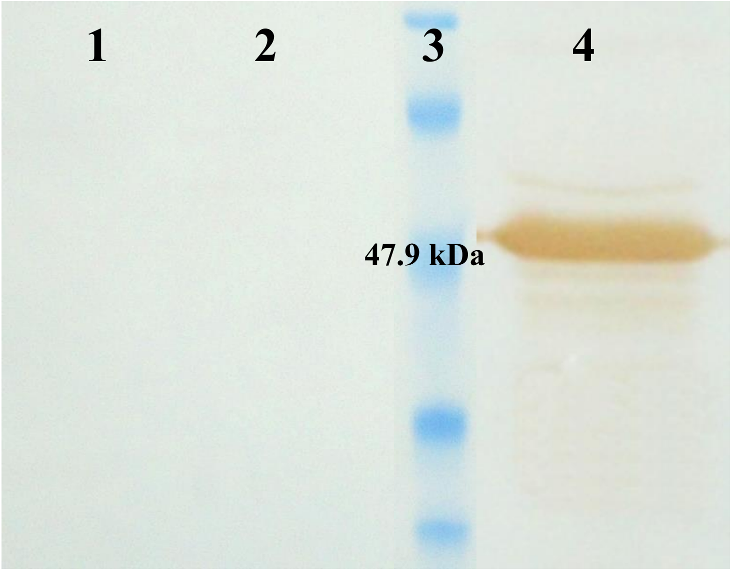

Supplemental Figure 3: Immunodetection of the *R. jostii* ADP-Glc PPase using antibodies raised against *M. tuberculosis* ADP-Glc PPase. Soluble fraction of control cells transformed with GroES-GroEL chaperone system and pET28c without *RjoglgC* insert (lane 1); sample corresponding to the control cells soluble fractions after capture with Ni-IDA resin (lane 2). Molecular mass marker (lane 3); sample corresponding to the soluble fraction of crude extracts from co-transformed *E. coli* cells (GroES-GroEL chaperone system plus [pET28c/*RjoglgC*] construction) after capture with Ni-IDA resin (lane 4).

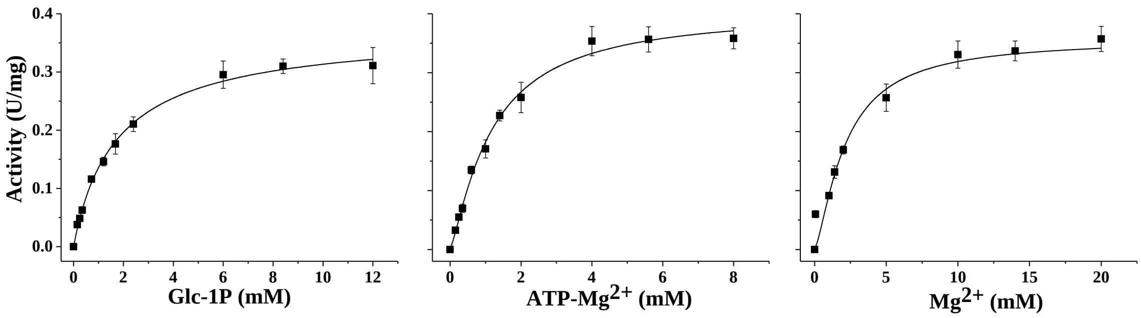

<u>Supplemental Figure 4</u>: *R. jostii* ADP-Glc PPase saturation curves for substrates. For experimental information see Materials and methods section and Supplementary Material 2.

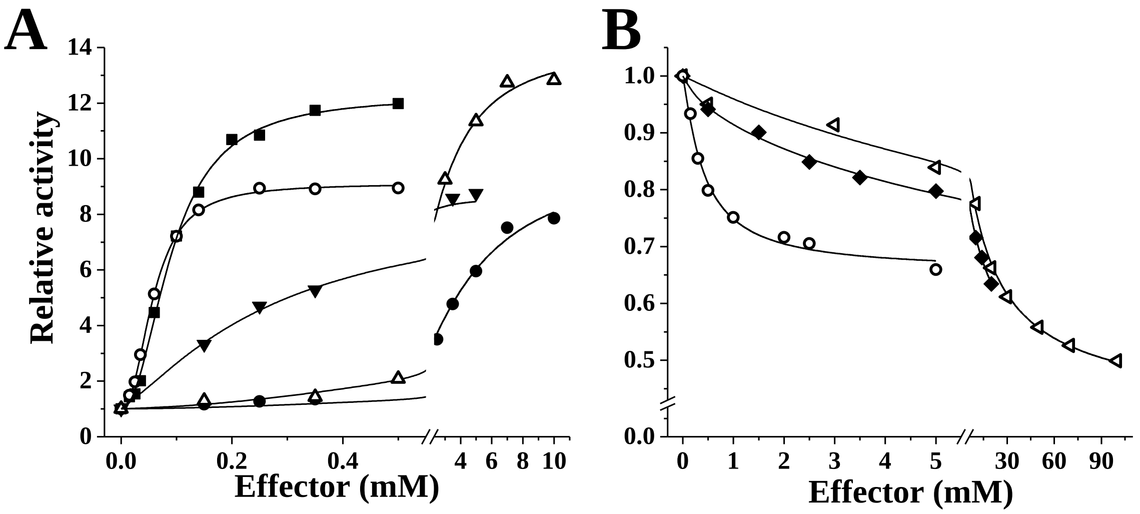

<u>Supplemental Figure 5</u>: Saturation curves of the *R. jostii* ADP-Glc PPase effectors. The value of 1 is the *R. jostii* ADP-Glc PPase V_{max} (0.39 ± 0.01 U/mg). **A:** saturation curves of the activators Glc-6P (filled square), Man-6P (open circle), PEP (filled triangles), Fru-6P (open triangles) and Rib-5P (filled circles). **B:** saturation curves of the *R. jostii* ADP-Glc PPase inhibitors Pyr (open triangles), NADPH (open circles) and 6-PGlcA (filled squares). For experimental information see Materials and methods section and Supplementary material 2.

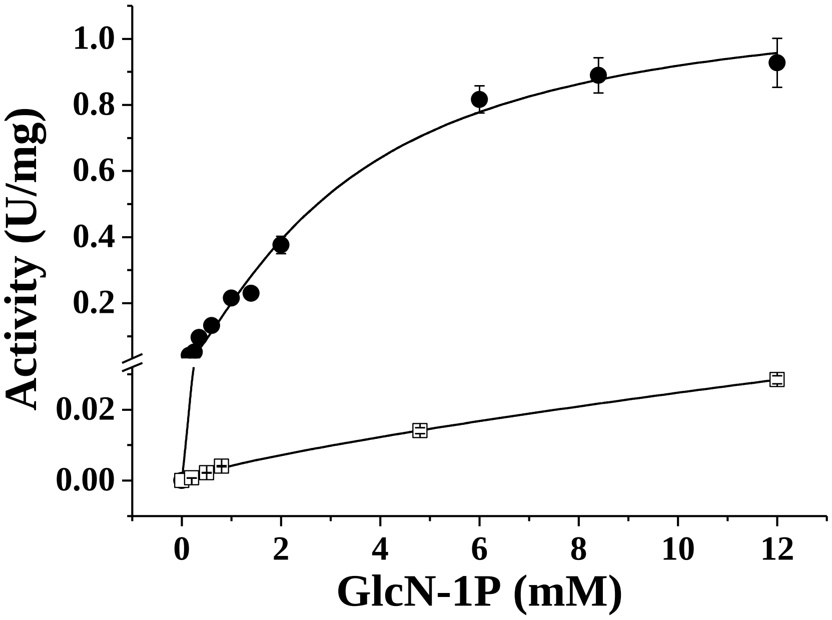

<u>Supplemental Figure 6</u>: Saturation curves for GlcN-1P of *R. jostii* ADP-Glc PPase determined in absence (empty squares) or presence (filled circles) of 0.5 mM Glc-6P.
